# Consumption of High-Polyphenol Dark Chocolate Improves Endothelial Function in Individuals with Stage 1 Hypertension and Excess Body Weight

**DOI:** 10.1155/2012/147321

**Published:** 2012-11-08

**Authors:** Lívia de Paula Nogueira, Marcela Paranhos Knibel, Márcia Regina Simas Gonçalves Torres, José Firmino Nogueira Neto, Antonio Felipe Sanjuliani

**Affiliations:** ^1^Discipline of Clinical and Experimental Pathophysiology, Rio de Janeiro State University, 20551-030 Rio de Janeiro, RJ, Brazil; ^2^Department of Applied Nutrition, Nutrition Institute, Rio de Janeiro State University, 20551-030 Rio de Janeiro, RJ, Brazil; ^3^Lipids Laboratory, Rio de Janeiro State University, 20551-030 Rio de Janeiro, RJ, Brazil

## Abstract

*Background*. Hypertension and excess body weight are important risk factors for endothelial dysfunction. Recent evidence suggests that high-polyphenol dark chocolate improves endothelial function and lowers blood pressure. This study aimed to evaluate the association of chocolate 70% cocoa intake with metabolic profile, oxidative stress, inflammation, blood pressure, and endothelial function in stage 1 hypertensives with excess body weight. *Methods*. Intervention clinical trial includes 22 stage 1 hypertensives without previous antihypertensive treatment, aged 18 to 60 years and presents a body mass index between 25.0 and 34.9 kg/m^2^. All participants were instructed to consume 50 g of chocolate 70% cocoa/day (2135 mg polyphenols) for 4 weeks. Endothelial function was evaluated by peripheral artery tonometry using Endo-PAT 2000 (Itamar Medical). *Results*. Twenty participants (10 men) completed the study. Comparison of pre-post intervention revealed that (1) there were no significant changes in anthropometric parameters, percentage body fat, glucose metabolism, lipid profile, biomarkers of inflammation, adhesion molecules, oxidized LDL, and blood pressure; (2) the assessment of endothelial function through the reactive hyperemia index showed a significant increase: 1.94 ± 0.18 to 2.22 ± 0.08, *P* = 0.01. *Conclusion*.In individuals with stage 1 hypertension and excess body weight, high-polyphenol dark chocolate improves endothelial function.

## 1. Introduction

The endothelium has emerged as a key regulator of vascular homeostasis, acting as an active signal transducer for metabolic, hemodynamic, and inflammatory factors that modify the function and morphology of the vessel wall. Alterations in endothelial-cell function can precede the development of atherosclerotic changes and the progression of cardiovascular diseases [1]. Hypertension and excess body weight (body mass index (BMI) ≥25 kg/m^2^) are conditions with high prevalence, being important risk factors for endothelial dysfunction [2–5]. According to World Health Organization (2009) [3] high blood pressure is responsible for 13% of deaths globally and overweight and obesity are responsible for 5%. Hypertension and obesity are frequently associated, and the causal association between obesity and elevated blood pressure has since long been demonstrated [6].

 Lifestyle interventions, including diet, may affect endothelial function: high-fat diets impair endothelial function, and diets such as the Mediterranean diet are associated with improved endothelial function [7]. Recently, cocoa and cocoa-derived products such as chocolate with 70% or more cocoa (dark chocolate) have gained attention because of evidences that they lower blood pressure and improve endothelial function [8–14]. These beneficial effects have been frequently ascribed to flavonoids, a subgroup of the polyphenolic family of antioxidant chemicals, abundantly present in fruits, vegetables, red wine, teas and cocoa. Catechin and its isomer epicatechin are types of flavonoids with strong antioxidant properties. Cocoa contains high concentrations of epicatechin and has been noted to have antioxidant content that is two times higher than that of red wine and almost three times higher than that of green tea [15, 16].

There are several plausible mechanisms by which polyphenols may improve endothelial function and lower blood pressure. In addition to their antioxidant effects which are assumed to increase the biodisponibility of nitric oxide (NO), polyphenols have been shown to increase the formation of NO by endothelial NO synthase via increased calcium level and redox-sensitive activation of the phosphoinositide 3 (PI3)-kinase/Akt pathway. Polyphenols also (1) enhance the production of endothelium-derived hyperpolarizing factor (EDHF) and prostacyclin and (2) inhibit the synthesis of vasoconstrictors such as endothelin-1 and the angiotensin-converting enzyme [14, 17].

Recent studies have also demonstrated beneficial effects of dark chocolate or cocoa on insulin resistance [9, 18–20], lipid profile [21, 22], and inflammatory status [23]. However, the number of studies evaluating the effect of dark chocolate or cocoa on these cardiovascular risk factors is relatively low, and some authors did not find all these benefits [24, 25].

The effects of dark chocolate or cocoa have already being evaluated in hypertensive individuals [9, 10, 19]. However, there is a lack of studies evaluating its effect in hypertensive individual presenting overweight and obesity. Therefore, the aim of the present study was to association of chocolate 70% cocoa intake with metabolic profile, oxidative stress, biomarkers of inflammation, blood pressure and endothelial function in stage 1 hypertensive subjects with excess body weight.

## 2. Materials and Methods

This pre-post trial was conducted at the Laboratory of Clinical and Experimental Pathophysiology, CLINEX, located at Pedro Ernesto University Hospital, of Rio de Janeiro State University. Written informed consent was obtained from all the enrolled patients. The study protocol was approved by the Human Ethics Committee of Pedro Ernesto University Hospital. The procedures followed in this study were in accordance with institutional guidelines.

Potential participants were recruited at the Department of Plastic Surgery, among the candidates for lipoplasty. Candidates for the study, underwent eligibility screening by registered dietitians. Subjects were screened according to the following criteria: age between 18 and 60 years, BMI from 25.0 to 34.9 kg/m^2^ and diagnosis of stage 1 hypertension (without previous antihypertensive treatment) [26]. The exclusion criteria were current use of antioxidant and dietary supplements; use of any medication known to interfere in body weight, blood pressure, and metabolic profile; recent changes (within previous 6 months) in dietary intake, body weight (>3 kg), and intensity or frequency of physical exercise. Individuals with eating disorders, major depression, or a medical history of drug addiction were excluded. Those with any metabolic disease, such as diabetes mellitus or hypothyroidism or chronic diseases severely affecting the cardiovascular, gastrointestinal, and renal systems were also excluded. Pregnant or lactating women were not allowed into the study.

**Figure 1:**
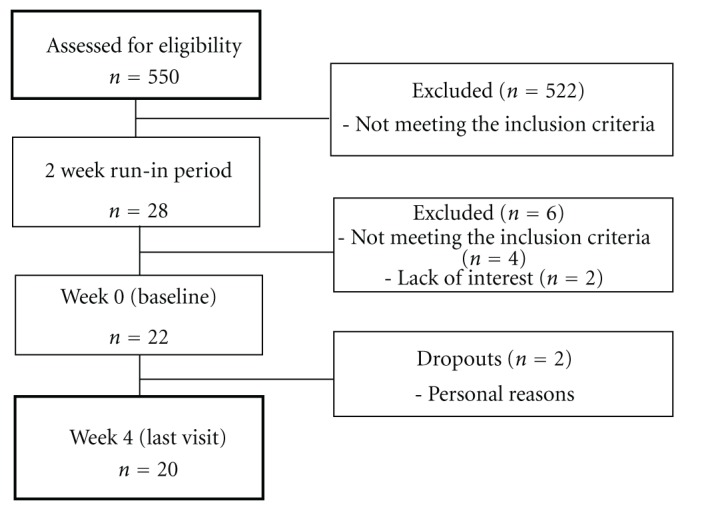
Flow diagram of the study.

Of the 550 individuals initially screened, 28 entered the run-in period. During the 2-week run-in period all cocoa foods were excluded, and potential participants were submitted to clinical, dietary, anthropometric, and biochemical evaluation. Six subjects failed to complete the run-in period, four individuals because their levels of blood pressure were not in the range of stage 1 hypertension and two because of lost of interest. So after completing the run-in period, 22 participants were included in the study and 20 completed the follow-up period (4 weeks), being included in the final analysis (Figure 1). The noncompleters left the study because of scheduling conflicts.

During the intervention phase (4 weeks), participants were submitted to clinical and nutritional assessment at baseline (week 0) and weeks 1, 2, 3, and 4. All participants were instructed to consume 50 g of chocolate 70% cocoa/day (containing 2135 mg polyphenols) for 4 weeks (Table 1). They received instructions to consume 25 g in the morning and 25 g in the afternoon. At baseline and at every week participants received the amount of chocolate sufficient for 7 days of the study. The development and reinforcement of strategies for continued success were made at the same time points.

Body weight, waist circumference, hip circumference were measured at baseline and at weeks 1, 2, 3, and 4. At baseline and at week 4 participants were also submitted to ambulatory blood pressure monitoring (ABPM) and evaluation of endothelial function; body composition and fasting plasma levels of circulating insulin, glucose, leptin, lipid profile (triglycerides, total cholesterol, high-density lipoprotein (HDL) cholesterol, and low density lipoprotein (LDL) cholesterol), biomarkers of inflammation (C-reactive protein, interleucina-6, and tumor necrosis factor-*α*), biomarker of oxidative stress (oxidized LDL), and biomarkers of endothelial dysfunction (intracelluar adhesion molecule-1 (ICAM-1), vascular cell adhesion molecule-1 (VCAM-1) and E-selectin). 

The dietary assessments during the run-in period used a 3-day food record, covering 2 weekdays, and 1 weekend day, to estimate current energy and nutrients consumption. To avoid weight gain during the study period, patients were instructed to reduce their habitual energy intake proportionally to the energy supplied by the chocolate (280 Kcal/day; mainly from lipid-rich foods). During the intervention phase, the food records were reviewed and clarified in an interview with a nutritionist every week to assess dietary adherence. Nutrient analysis of the 3-day food record was performed using the software NutWin (São Paulo Federal University, UNIFESP, São Paulo, Brazil). Reported total energy, percent of energy from protein, fat and carbohydrate, and fiber content at the run-in phase were similar to that of the last week of the study. Subjects were carefully instructed to refrain from flavonoid-rich foods and beverages, including tea and wine; a list of these foods and beverages was given to each participant. All participants were encouraged to continue their usual physical activity throughout the study period.

Height, weight, and waist and hip circumferences were measured from 08:00 to 10:00 h after a 12 h fast. Height was measured using a stadiometer accurate to ±0.5 cm, and weight was obtained with a calibrated scale, accurate to ±0.1 kg (Filizola S.A., São Paulo, SP, Brazil), with participants wearing light clothing and no shoes. BMI was calculated using the standard equation (kilograms per meters squared). Waist circumference was measured in the standing position, midway between the lower margin of the last rib and the iliac crest. The measurements were taken at midexhalation. Hip circumference was measured at the widest point of the hip/buttocks area with the measuring tape parallel to the floor. Waist-to-hip ratio was determined by dividing waist circumference by hip circumference. Anthropometric measurements were taken twice, and mean values were used in all analyses. Percentage of body fat was estimated by electrical bioimpedance using a Biodynamics BIA-450 body fat analyzer (Biodynamics Corp., Seattle, WA, USA).

Blood samples were collected after a 12 h fasting period and were stored at −80°C. Total cholesterol, HDL cholesterol, and triglyceride concentrations were assessed by using an automated analyzer. LDL cholesterol was calculated using the Friedewald formula [27] when triglycerides did not exceed 400 mg/dL. Radioimmunoassay was used to determine plasma leptin and insulin levels (Linco Research, St Charles, MO, USA, double antibody solid-phase enzyme immunoassay). Fasting plasma glucose was determined by the use of glucose oxidase method. The insulin resistance status was assessed by the use of homeostasis model assessment of insulin resistance (HOMA-IR) index, that is, serum insulin (*μ*U/mL) × plasma glucose (mmol/L)/22.5 [28]. The values of VCAM-1, ICAM-1 and E-Selectin were determined by immunonephelometry enzymatic immunoassay using a commercial kit of multiple dosing (LINCO Research, St Charles, MO, USA). Plasma levels of TNF-*α* and IL-6 were determined by enzymatic immunometric method (TiterZyme EIA) using commercial kits (Assay Designs, Ann Arbor, MI, USA). Highly sensitive CRP (hs-CRP) was determined by turbidimetry, using commercial kit (Biosystems, Barcelona, Spain). 

### 2.1. Endothelial Function

 Endothelial function was evaluated by peripheral artery tonometry (PAT) using Endo-PAT 2000 (Itamar Medical, Caesarea, Israel), a finger plethysmographic device that allows the isolated detection of pulsatile arterial volume changes [29]. Endo-PAT 2000 is a noninvasive technology and was approved by the Food and Drug Administration for use as a diagnostic aid in patients with signs and symptoms of ischemic heart disease [30]. There is evidence of a significant relationship between hyperemia-induced finger pulse wave amplitude changes, defined as the PAT hyperemia ratio, and brachial artery flow-mediated dilation [31].

Endo-PAT consists of two finger-mounted probes, which include a system of inflatable latex air cushions within a rigid external case. The probe design allows the application of a constant and evenly distributed near-diastolic counterpressure within the entire probe, which increases sensitivity by unloading arterial wall tension and prevents venous blood pooling to avoid venoarteriolar reflex vasoconstriction. Pulsatile volume changes of the fingertip are sensed by a pressure transducer and transferred to a personal computer where the signal is band pass filtered (0.3 to 30 Hz), amplified, displayed, and stored [29]. The Endo-PAT studies were performed with the patient in the supine position and both hands on the same level in a comfortable, thermoneutral environment. A blood pressure cuff was placed on one upper arm (study arm), while the contralateral arm served as a control (control arm); Endo-PAT probes were placed on one finger of each hand (same finger on both hands). A continuous recording of pulsatile blood volume responses from both hands was initiated. After a 10 min equilibration period, the blood pressure cuff on the study arm was inflated to 60 mmHg above systolic pressure for 5 min. The cuff was then deflated to induce reactive hyperemia, whereas PAT recording was continued. The reactive hyperemia index (RHI) obtained with Endo-PAT is analyzed by a computer in an operator-independent manner. 

### 2.2. Blood Pressure

During the run-in phase blood pressure was recorded by using a calibrated Dinamap 1846 Critikon automated sphygmomanometer (Critikon, Tampa, FL, USA) after a resting period of at least 10 min in the sitting position. An appropriate arm cuff was used. Arm position was adjusted so that the cuff was at the level of the right atrium. Blood pressure was measured on the nondominant arm every 3 min for 15 minutes. The first value was discarded, and the mean of the last five readings was used in the analysis. Patients were considered to have stage 1 hypertension if their systolic blood pressure levels were between 140–159 mmHg and/or diastolic blood pressure between 90–99 mmHg [26].

At baseline (week 0) and at the end of the study (week 4) blood pressure was evaluated by Ambulatory Blood Pressure Monitoring (ABPM) to improve the estimate of “true” blood pressure. Ambulatory blood pressure was recorded using the SpaceLabs 90207 oscillometric blood pressure monitors (SpaceLabs, Redmond, WA, USA) calibrated against a mercury sphygmomanometer before use on each patient. Monitors were programmed to read blood pressure and heart rate every 20 min from 6:00 to 18:00 h. and every 30 min from 18:00 to 6:00 h. Mean daytime (6:00 to 18:00 h), and nighttime (18:00 to 6:00 h) blood pressure and heart rate were calculated.

### 2.3. Statistical Analysis

Means ± standard errors were used to summarize continuous variables. To test the possible association of chocolate 70% cocoa intake with nutritional parameters, biochemical variables, endothelial function, and blood pressure, we compared data obtained at baseline (week 0) with data obtained at the end of the study (week 4). Paired Student's *t*-test was used when variables had normal distribution and Wilcoxon test was used for variables without normal distribution. 

Correlation tests were conducted to determine the relationship between RHI and variables of interest. Partial correlations controlled for different confounders were also used.

On the basis of a previous study [9], this trial was designed to have 80% power to detect a significant difference in systolic blood pressure evaluated by ABPM before and after the intake of dark chocolate. Assuming 20% dropout rate, we needed at least 12 participants in the study. GraphPad PRISM 5.0 (GraphPad Software Inc, San Diego, CA, USA) and Stata 10.0 (STATA Corp., College Station, TX, USA) were used for statistical analysis. *P* < 0.05 was considered statistically significant. 

## 3. Results

 Twenty participants (10 men and 10 women) completed the study and were included in the final analysis. The average age of these patients was 44.00 ± 2.87 years, and their BMI was 31.29 ± 1.16 kg/m^2^. During the run-in phase, systolic and diastolic blood pressure levels were 146.50 ± 1.28 and 93.20 ± 0.74 mmHg, respectively. 

As expected from the experimental design, all anthropometric parameters and the percentage of body fat remained almost unchanged after the 4 weeks of chocolate supplementation (Table 2). As presented in Table 3, at the end of the study, there were no significant changes in glucose metabolism, lipid profile, biomarkers of inflammation, and oxidized LDL. 

**Figure 2:**
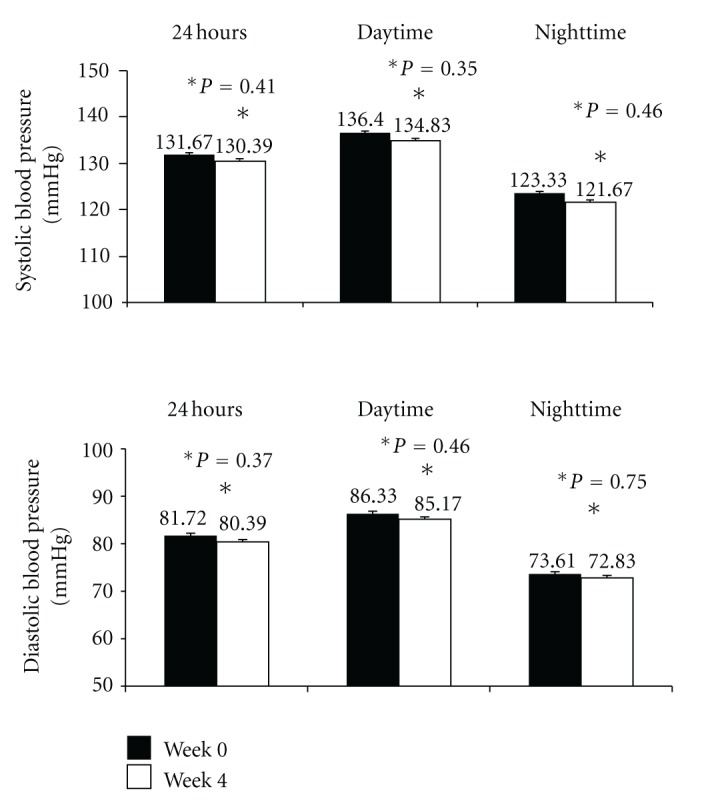
Mean values of systolic and diastolic blood pressure evaluate by ambulatory blood pressure monitoring at baseline (week 0) and at the end of the study (week 4) (*n* = 20).

The comparative analysis of the values obtained with ABPM between week 0 and week 4 revealed no significant modifications. However, there was a small decrease in both systolic and diastolic blood pressure at 24 h, daytime and nighttime (Figure 2).

**Figure 3:**
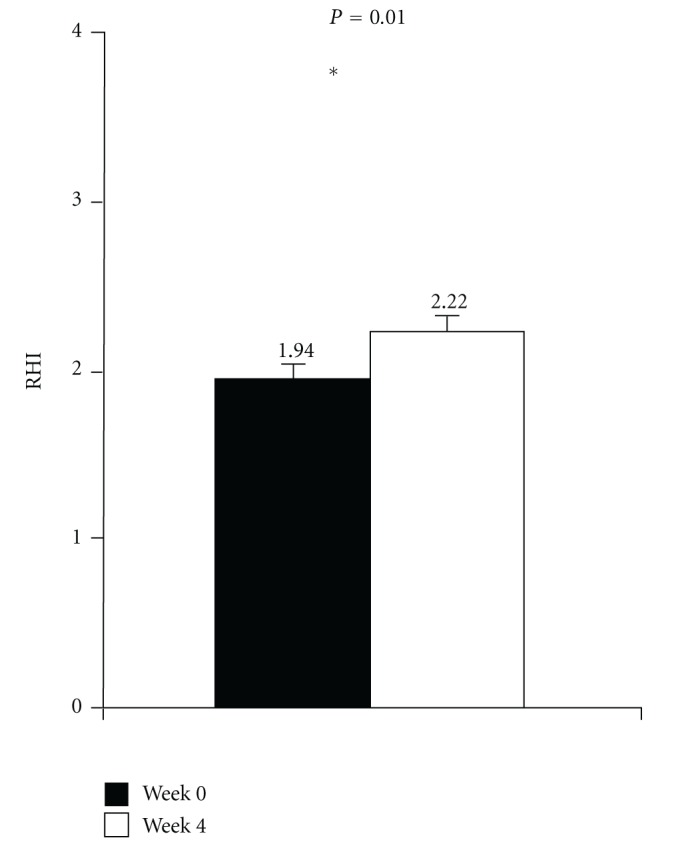
Mean reactive hyperemia index (RHI) evaluated by Endo-PAT2000 at baseline (week 0) and at the end of the study (week 4) (*n* = 20).

**Figure 4:**
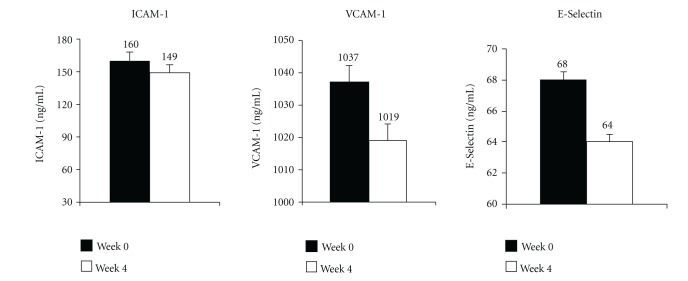
Mean values of intracellular adhesion molecule-1 (ICAM-1), vascular cell adhesion molecule-1 (VCAM-1), and E-selectin at baseline (week 0) and at the end of the study (week 4) (*n* = 20).

After 4 weeks of chocolate supplementation there was a significant increase in RHI (Figure 3). Serum levels of adhesion molecules, which are biomarkers of endothelial dysfunction, also showed a decrease, however, without reaching statistical significance (Figure 4). 

An inverse and a significant association was observed between the RHI at baseline and the modification in this index during the study period (*r* = −0.60; *P* = 0.02). Even after adjusting for confounders, this association remained significant (*r* = −0.70; *P* = 0.04). The confounding factors included in this analysis were age and changes during the study period in BMI, total cholesterol, HDL cholesterol, LDL cholesterol, triglycerides, hs-CRP, and HOMA.

The changes in RHI during the study presented a negative and significant association with the modifications in diurnal systolic and diastolic blood pressure (*r* = −0.69; *P* = 0.04 and *r* = −0.83; *P* = 0.006) after adjusting for age and changes in BMI, HOMA, and hs-CRP. Modifications in nocturnal and 24 h blood pressure did not present significant associations with changes in RHI. 

## 4. Discussion

In the present study, based on a sample of subjects with stage 1 hypertension and excess body weight, the main finding was that the consumption of high-polyphenol dark chocolate 70% cocoa (50 g/day, during four weeks) improved endothelial function. 

An improvement in endothelial function after high-polyphenol cocoa and/or dark chocolate intake was observed in several studies [9, 11, 12, 18, 19, 32–37]. These studies have different designs, varying principally in relation to the time and dose of supplementation and in relation to the criteria of eligibility of the participants. Even with different designs the studies found significant improvement in endothelial function that was evaluated in the great majority of the trials by flow-mediated vasodilatation of the brachial artery [9, 11, 12, 19, 34–36]. To our knowledge only one study [12] evaluated endothelial function using the same method that we used (pulse-wave amplitude on the finger assessed by PAT), although the device that was used in our study is a different one. 

The duration of the studies varies widely: there are trials that observe acute effects (in general 2 h after the intake of cocoa products) [18, 33–36], or short-term effects: 5 days [12], 2 weeks [9, 11, 19, 37], 4 weeks [38], 6 weeks [32] and 12 weeks [18]. The duration of the present study, although was not sufficient to evaluate the long-term effect of dark chocolate intake on endothelial function, it is greater than the duration of several studies. 

The participants of the clinical trials that observed improvements in endothelial function with cocoa supplementation included health individuals [11, 12, 37], hypercholesterolemic postmenopausal women [32], heart transplant recipients [33], smokers [35, 36], hypertensives [9], hypertensives with impaired glucose intolerance, [19], individuals with diabetes [38], and overweight and obese individuals [18]. As stated before, only individuals with hypertension and excess body weight were included in the present study, creating a difference from other studies and showing that in this population of high risk to endothelial dysfunction the supplementation of high-polyphenol dark chocolate alone (without specific treatment for hypertension and excess body weight) can improve endothelial function.

The participants of this study presented a decrease in blood pressure levels, although without reaching statistical significance. This finding contrasts with some studies that also evaluated the effects of cocoa and/or cocoa rich chocolate in hypertensive individuals [8–10, 19]. 

Desch et al. (2010) [8] performed a meta-analysis of randomized controlled trials assessing the antihypertensive effects of flavanol-rich cocoa products. In total 10 trials comprising 297 individuals were included. The populations studied were either healthy normotensive adults or patients with prehypertension/stage 1 hypertension. This meta-analysis confirmed the blood pressure lowering capacity of flavanol-rich cocoa [8]. However, in a recently published meta-analysis [39] newer studies were included, totalizing 15 trials and were performed a subgroup analysis by baseline blood pressure (hypertensive/normotensive). Pooled meta-analysis of all trials revealed a significant blood pressure-reducing effect of cocoa-chocolate compared with control. However, subgroup meta-analysis was significant only for the hypertensive or pre-hypertensive subgroups, while blood pressure was not significantly reduced in the normotensive subgroups [39]. 

Possible explanations for the lack of significant reduction in blood pressure in the present study are (1) baseline levels of blood pressure, evaluated by ABPM, in our patients were lower than the levels found in others studies and (2) dose of dark chocolate was also lower [9, 19]. Ried et al. (2009) [40] did not find a blood pressure reducing effect of 50 g dark chocolate daily over a period of 8 weeks in a prehypertensive population.

In the present study, the lipid profile had no significant modifications. However, some studies observed significant reductions on total and/or LDL cholesterol after the intake of dark chocolate or cocoa [19, 21, 22]. Mursu et al. (2004) [41] found that the ingestion of 75 g/day of dark chocolate for 15 days increased HDL cholesterol. The changes seen in lipid profile in the studies cited above were highly dependent on the dose of cocoa consumption and health status of patients [16]. The dose of dark chocolate used in the study of Mursu et al. (2004) [41] and Grassi et al. (2008) [19] was 75 g/day and 100 g/day, respectively. Therefore, one possible explanation for the significant changes in serum lipids on our study may be the dose of chocolate. Another possible explanation is that at baseline our participants had mean levels of lipid profile within the normal range according to NCEP (2001) [42].

The content of lipids in dark chocolate is high. As seen in Table 1, in the present study, the ingestion of 50 g/day of chocolate resulted in an intake of 20 g of total fat/day. Despite its high fat content, cocoa itself does not seen to exert untoward effects on serum lipids (and in some studies has beneficial effects), because cocoa butter is composed on average of 33% oleic acid, 25% palmitic acid, and 33% of stearic acid [43]. Oleic acid is a monounsaturated fat that lowers LDL cholesterol [42] and although palmitic and stearic acids are saturated fats, stearic acid in comparison with other saturated fatty acids lowers LDL cholesterol [44, 45].

All participants included in the present study had excess body weight (BMI from 25.0 to 34.9 kg/m^2^). As weight loss improves endothelial function and decreases blood pressure, while weight gain has the opposite effect [46–48]; an important issue of our study was to instruct the participants to maintain their body weight. It is important to notice that all the patients included in our study did not present recent changes in body weight before the study and were not planning to begin an energy restricted diet or to increase physical activity. After the end of the study all participants were scheduled to a consultation with a nutritionist and with a physician to begin the treatment for hypertension and weight loss. 

There are several limitations in our study. One of them is the lack of a control group, which was not planned in the initial design of the study. However, the eligibility criteria of the present study were to rigorously to try to avoid confounding factors, so as seen in Figure 1 we assessed for eligibility 550 individuals and only 22 could be included in the study.

## 5. Conclusion

The findings of the present study suggest that, in individuals with stage 1 hypertension and excess body weight, the consumption of high-polyphenol dark chocolate is associated with improvements in endothelial function.

## Figures and Tables

**Table 1:** Nutrient composition of the dark chocolate used in the study (50 g).

Nutrient	Dark chocolate (70% cocoa)
Energy (kcal)	228
Protein (g)	4.8
Carbohydrate (g)	20
Total fat (g)	20
Saturated fat (g)	13.4
Sodium (mg)	60

**Table 2:** Anthropometric parameters and percentage of body fat at baseline (week 0) and at the end of the study (week 4).

Characteristic	Week 0 (*n* = 20)	Week 4 (*n* = 20)	*P*
Body weight (kg)	84.80 ± 3.88	84.63 ± 3.91	0.55
Body mass index (kg/m^2^)	31.29 ± 1.16	31.26 ± 1.19	0.83
Waist circumference (cm)	94.30 ± 2.73	94.07 ± 2.83	0.59
Hip circumference (cm)	110.70 ± 2.50	110.65 ± 2.49	0.85
Waist-to-hip ratio	0.85 ± 0.14	0.85 ± 0.15	0.79
Body fat (%)	36.59 ± 1.45	36.46 ± 1.41	0.69

Values are expressed as mean ± standard error.

**Table 3:** Metabolic variables and biomarkers of inflammation and oxidative stress at baseline (week 0) and at the end of the study (week 4).

Variable	Week 0 (*n* = 20)	Week 4 (*n* = 20)	*P*
Glucose (mg/dL)	90.60 ± 2.60	88.65 ± 2.75	0.55
Insulin (*μ*U/mL)	21.86 ± 3.11	23.49 ± 2.94	0.19
HOMA-IR	4.94 ± 0.79	5.08 ± 0.63	0.20
Total cholesterol (mg/dL)	199.00 ± 7.41	195.15 ± 9.25	0.55
HDL cholesterol (mg/dL)	50.85 ± 2.31	48.75 ± 2.64	0.43
LDL cholesterol (mg/dL)	122.15 ± 6.71	122.00 ± 9.24	0.98
Triglycerides (mg/dL)	132.80 ± 11.18	122.55 ± 11.77	0.29
High sensitive CRP (mg/dL)	0.93 ± 0.27	0.61 ± 0.12	0.24
Tumor necrosis factor-*α* (pg/mL)	17.51 ± 8.03	18.96 ± 9.02	0.18
Interleucine-6 (pg/mL)	87.87 ± 20.6	69.40 ± 14.7	0.17
Oxidized LDL (*μ*g/mL)	0.11 ± 0.01	0.12 ± 0.01	0.60

Values are expressed as mean ± standard error.

HDL: high-density lipoprotein, LDL: low density lipoprotein, CRP: C-reactive protein.
